# Identification of lipid metabolism-related gene signature in the bone marrow microenvironment of multiple myelomas through deep analysis of transcriptomic data

**DOI:** 10.1007/s10238-024-01398-w

**Published:** 2024-06-25

**Authors:** Dan Feng, Zhen Wang, Shengji Cao, Hui Xu, Shijun Li

**Affiliations:** 1 Department of Clinical Laboratory, The First Affiliated Hospital of Dalian Medical University, Liaoning Dalian, 116011 China; 2 Department of Clinical Laboratory, Harbin Medical University Cancer Hospital, Harbin, 150000 Heilongjiang China; 3 College of Laboratory Medicine, Dalian Medical University, Liaoning Dalian, 116044 China

**Keywords:** Multiple myeloma, Bone marrow microenvironment, Lipid metabolism-related genes, Prognosis, ACBD6, Machine learning, Immune infiltration

## Abstract

Dysregulated lipid metabolism in the bone marrow microenvironment (BMM) plays a vital role in multiple myeloma (MM) development, progression, and drug resistance. However, the exact mechanism by which lipid metabolism impacts the BMM, promotes tumorigenesis, and triggers drug resistance remains to be fully elucidated.By analyzing the bulk sequencing and single-cell sequencing data of MM patients, we identified lipid metabolism-related genes differential expression significantly associated with MM prognosis, referred to as LMRPgenes. Using a cohort of ten machine learning algorithms and 117 combinations, LMRPgenes predictive models were constructed. Further exploration of the effects of the model risk score (RS) on the survival status, immune status of patients with BMM, and response to immunotherapy was conducted. The study also facilitated the identification of personalized therapeutic strategies targeting specified risk categories within patient cohorts.Analysis of the scRNA-seq data revealed increased lipid metabolism-related gene enrichment scores (LMESs) in erythroblasts and progenitor, malignant, and Tprolif cells but decreased LMESs in lymphocytes. LMESs were also strongly correlated with most of the 50 hallmark pathways within these cell populations. An elevated malignant cell ratio and reduced lymphocytes were observed in the high LMES group. Moreover, the LMRPgenes predictive model, consisting of 14 genes, showed great predictive power. The risk score emerged as an independent indicator of poor outcomes. Inverse relationships between the RS and immune status were noted, and a high RS was associated with impaired immunotherapy responses. Drug sensitivity assays indicated the effectiveness of bortezomib, buparlisib, dinaciclib, staurosporine, rapamycin, and MST-312 in the high-RS group, suggesting their potential for treating patients with high-RS values and poor response to immunotherapy. Ultimately, upon verification via qRT-PCR, we observed a significant upregulation of ACBD6 in NDMM group compared to the control group.Our research enhances the knowledge base regarding the association between lipid metabolism-related genes (LMRGs) and the BMM in MM patients, offering substantive insights into the mechanistic effects of the BMM mediated by LMRGs.

**Graphical abstract:**

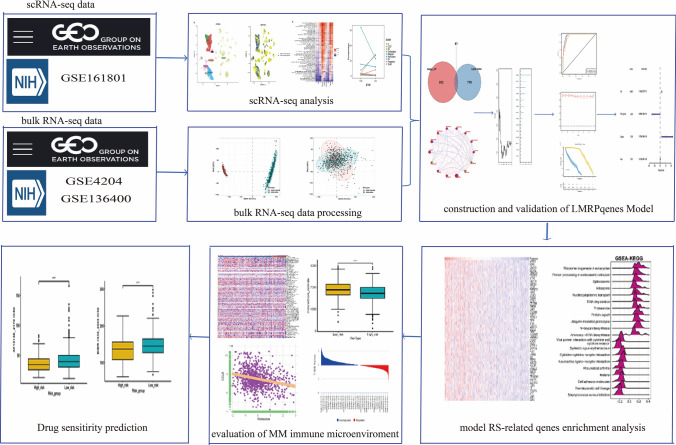

## Introduction

Multiple myeloma (MM) is an incurable hematological malignancy marked by the extensive proliferation and aggregation of monoclonal plasma cells (PCs) within the bone marrow (BM). This tumor type is typically aggressive and has a steadily increasing incidence rate. Currently, it has become the second most prevalent hematological tumor [1]. The application of proteasome inhibitors (PIs), immunomodulators, monoclonal antibodies (mAbs), and chimeric antigen receptor T cell (CAR-T) immunotherapy has significantly enhanced the survival prospects of MM patients, increasing the 5-year survival rate from 25 to over 50% [2]. Despite these advances, the phenomenon of drug resistance remains a significant hurdle, and the underlying mechanisms of MM drug resistance remain largely elusive. Multiple factors are believed to contribute to the development of drug resistance, such as genetic mutations in crucial drug targets, clonal evolution of malignant cells, and alterations within the BMM, among which the "natural selection" of the BMM is crucial for MM drug resistance [3–5].

The BMM, a complex milieu comprising cellular and acellular components, is crucial for MM cell survival and proliferation. It regulates host nutrition, metabolism, and immune status and participates in the pathophysiology of various tumors, including breast cancer, prostate cancer, and MM [6–9]. In particular, BM adipocytes (BMAds), which constitute approximately 70% of the BM volume in MM patients, are integral to the BMM architecture. Recent investigations have highlighted the critical role of metabolic reprogramming, driven by alterations in MM lipid metabolism, in supporting energy production, membrane biosynthesis, and lipid-mediated signaling pathways during cancer progression [10–12]. Additionally, the accumulation of lipid droplets in malignant PCs represents an adaptive mechanism crucial for their survival under adverse conditions [13].

Thus, understanding and targeting lipid metabolic pathways in MM has emerged as a promising therapeutic strategy for treating this malignancy.

## Materials and methods

### Data collection and preprocessing

Sequencing data and corresponding clinical data from the single-cell sequencing dataset (scRNA-seq) (GSE161801) [5] were obtained from the TISCH database (http://tisch.comp-genomics.org/home/) [14]. The analysis focused on BM samples from 19 relapsed refractory multiple myeloma (RRMM) patients before treatment. Additionally, the bulk RNA-seq datasets GSE4204 [15] (with 559 pretreatment samples) and GSE136400 [16] (with 354 pretreatment samples) were obtained from the Gene Expression Omnibus (GEO) database (https://www.ncbi.nlm.nih.gov/geo/). Using R “limma” and “sva” to remove batch effects, a merged dataset of 892 samples and 16,729 genes was prepared for further analysis. Lipid metabolism-related genes (LMRGs) were retrieved from the MsigDB by searching (https://www.gsea-msigdb.org/gsea/msigdb/human/geneset/REACTOME_METABOLISM_OF_LIPIDS and https://www.gsea-msigdb.org/gsea/msigdb/human/geneset/WP_LIPID_METABOLISM_PATHWAY), and a total of 760 LMRGs were obtained after merging and deduplication.

### Analysis of scRNA-seq data

The scRNA-seq data were analyzed with the R package “Seurat.” Filtration and cell identification were carried out directly using the filtration conditions of the TISCH database. The unified modular approximation and projection (UMAP) algorithm was applied for downscaling visualization and cell clustering annotation on the single-cell data. Then, nine distinct cell clusters were identified: B, CD8 + T, dendritic (DC), erythroblast, malignant, natural killer (NK), monocyte/macrophage, progenitor, and Tprolif cell clusters.

Differentially expressed genes (DEGs) across various cell types were pinpointed employing the “FindAllMarkers” function of the R “Seurat.” The functions highlighted the top 5 DEGs exhibiting high and low expression levels within each cell cluster. Marker genes for each cell type were identified by the R “COSG,” and a heatmap was generated to visualize the top 10 DEGs.

Furthermore, according to the “COSG” analysis results, the top 100 genes from each cell cluster were selected and subsequently enriched using the R “clusterprofiler.” This enrichment included KEGG and Reactome pathway analyses.

### Correlation analysis between the expression of lipid metabolism-related genes and hallmark gene sets

Information on 50 hallmark pathways was retrieved from the MsigDB (http://www.gsea-msigdb.org/gsea/msigdb/index.jsp). Lipid metabolism-related gene enrichment scores (LMESs) for each cell, along with the enrichment score of each hallmark pathway for each cell, were calculated employing “ssGSEA” from the R package “GSVA.” To identify potential pathways associated with lipid metabolism, we performed a correlation analysis between LMES and the 50 hallmark pathways, with the findings depicted in a heatmap. Subsequently, the samples were divided into high and low LMES groups according to the median expression of LMRGs, followed by an analysis of cell distribution between the two groups.

### Lipid metabolism-related DEGs with potential prognostic value

Differential analysis was performed by comparing normal and tumor specimens of single-cell data (logFC > 0.25 and *p* < 0.05). This analysis revealed 974 genes with increased expression and 372 genes with decreased expression by adjusting *p* value. These DEGs, both upregulated and downregulated, intersected with 760 LMRGs, identifying 41 genes whose expression increased and 7 whose expression decreased differentially related to lipid metabolism (LMR-DEGs). To pinpoint LMR-DEGs with potential prognostic significance, Spearman correlation, univariate Cox regression, and survival analyses were conducted using the R “survival” and “survminer” R packages for the 48 LMR-DEGs and the R “ggplot2” R package for visualization. Ultimately, 14 genes (COX *p* < 0.05) demonstrating prognostic potential, referred to as LMRPgenes, were selected for inclusion in the prognostic model.

### Predictive model construction based on LMRPgenes by multiple machine learning methods

The GSE4204 and GSE136400 datasets were merged as the training set. Ten classic algorithms were integrated to construct a predictive model. These include random forest (RSF), least absolute shrinkage and selection operator (LASSO), gradient boosting machine (GBM), survival support vector machine (Survival-SVM), supervised principal component (SuperPC), ridge regression, partial least squares regression for Cox (plsRcox), CoxBoost, stepwise Cox, and elastic network (Enet) methods. To form 117 machine learning combinations, RSF, LASSO, CoxBoost, and stepwise Cox were combined with other algorithms. These four algorithms are known for their ability to reduce dimensionality and screen variables. The concordance index (C-index) and average C-index were calculated to evaluate the predictive power of the different algorithms and to identify the optimal predictive model. The model derived by RSF emerged as the optimal model. Employing this model, a risk score (RS) was obtained.

The samples were categorized into high- and low-RS groups depending on the RS cutoff value used for survival analysis. Survival analysis was conducted to explore the variation in survival status across different RS groups within the GSE4204, GSE136400, and merged datasets.

### Evaluation of predictive models based on LMRPgenes

In this study, the R “pROC” was used to generate receiver operating characteristic (ROC) curves to evaluate the diagnostic performance of the model across three datasets at 1-, 3-, and 5-year intervals for MM. The area under the curve (AUC) was computed to determine the diagnostic efficacy of the model. Moreover, the “plotAUCcurve” function within the R “timeROC” facilitated the plotting of time-dependent AUC variations, enabling an assessment of the model’s predictive accuracy over time. The R package “survival” was used to construct survival curves to examine the potential disparities in overall survival (OS) between the high- and low-RS groups. Additionally, the relationships between age, sex, and the RS were investigated. We performed univariate and multivariate Cox regression analyses on the three datasets to determine whether the RS was an independent prognostic factor for predicting OS in MM patients.

### Enrichment analysis of RS-related genes

We performed a correlation analysis between RS and all genes. A heatmap was created to visualize the top 50 genes displaying positive and negative correlations with the RS. Subsequently, a gene set enrichment analysis (GSEA) of these identified genes was conducted employing the R "clusterProfiler." This analysis included GO, KEGG, and Reactome pathway analyses.

### Correlation analysis of immune characteristics in BMM with model RS

Recently, there have been significant advancements in immunotherapy and targeted treatments for MM patients. To explore the relationship between the model RS and the immune microenvironment of MM patients, we employed the R "IOBR" to assess the BM immune status of MM patients. We employed eight distinct algorithms[17], including microenvironment cell populations-counter (MCPcounter), estimating the proportion of immune and cancer cells (EPIC), xCell, cell-type identification by estimating relative subsets of RNA transcripts (CIBERSORT), QuanTIseq, tumor immune estimation resource (TIMER), immunophenoscore (IPS), and ESTIMATE. ESTIMATE was applied to compare the immune, stromal, ESTIMATE, and tumor purity scores between the high- and low-RS groups. Furthermore, by employing the IPS, we calculated the scores for four diverse immunophenotypes: antigen presentation, effector cells, suppressor cells, and checkpoints. The MCPcounter was used to analyze immune cell infiltration between the two groups. Subsequent analyses with other algorithms corroborated these findings. Finally, the correlation between the RS and cytokines, along with their receptors, was evaluated.

### Evaluation of immunotherapy efficacy and sensitivity drug prediction

Due to the complexity of the microenvironment in MM, the assessment of immune status mentioned above falls short of adequately reflecting the effectiveness of immunotherapy. Consequently, we used the tumor immune dysfunction and exclusion (TIDE) database to predict immunotherapy outcomes and provide related values (TIDE values).

The TIDE value serves as a barometer for the success of immunotherapy, where a higher value indicates diminished efficacy. Additionally, we employed R "OncoPredict" to predict the efficacy of various nonimmunotherapy anticancer drugs.

By determining the IC50 values for each anticancer drug, we can gauge their potency and discern the difference between the high- and low-RS groups. Notably, an elevated IC50 value signifies reduced sensitivity to the drug.

### Tissue Sample Collection

Bone marrow (BM) samples, including 22 NDMM patients, 9 healthy individuals (control group), and 5 MM patients in complete remission (CR group), were collected by BM aspiration at the First Affiliated Hospital of Dalian Medical University. BM samples were transported to the laboratory within two hours for processing. BM slides were analyzed by three specialists who quantified the percentage of plasma cells and determined the mean values. Single nucleated cells were isolated from these samples and preserved at -80°C until further analysis. Ethical clearance for this study was granted by the Research Ethics Review Committee of the First Affiliated Hospital of Dalian Medical University, and informed consent was obtained from all participants.

### Quantitative Real-Time Reverse Transcription Polymerase Chain Reaction (qRT-PCR)

For the qRT-PCR analysis, total RNA was extracted from the single nucleated cell samples using a Trizol RNA isolation reagent. The RNA was then reverse-transcribed into cDNA using the PrimeScript RT Reagent Kit (Takara, DRR037A). Quantification of RNA expression was conducted using the SYBR Premix Ex Taq II (Takara) on an ABI 7500 real-time PCR system. The relative expression levels were measured using the comparative Ct method, with gene expression levels normalized to GAPDH. Data were presented as mean ± SD from three replicates. The primer sequences used in amplification were as follows: Forward, 5′-GGCCTGTGATCGAGGACATAA-3′; Reverse, 5′-GCAGCAGCTCTACAATATCCAGAA-3′.

### Statistical analysis

All the statistical analyses were conducted with R software (R 4.2.1). The analysis of the scRNA-seq data was facilitated using the R "Seurat." R "survival" and "survminer" were used to perform the Kaplan‒Meier (K–M) survival analysis. Univariate and multivariate Cox regression analyses were performed to identify independent prognostic indicators. The performance of the model was assessed by plotting the ROC curve and calculating the AUC using the "pROC" R package. Spearman's method was utilized to perform correlation analyses. The Kruskal–Wallis test was utilized as a nonparametric method to estimate gene expression differences among three groups. *p* values less than 0.05 were considered to indicate statistical significance and are annotated as follows: *p < 0.05; **p < 0.01; ***p < 0.001; ****p < 0.0001.

## Results

### Results of single-cell sequencing analysis

**Figure 1:**
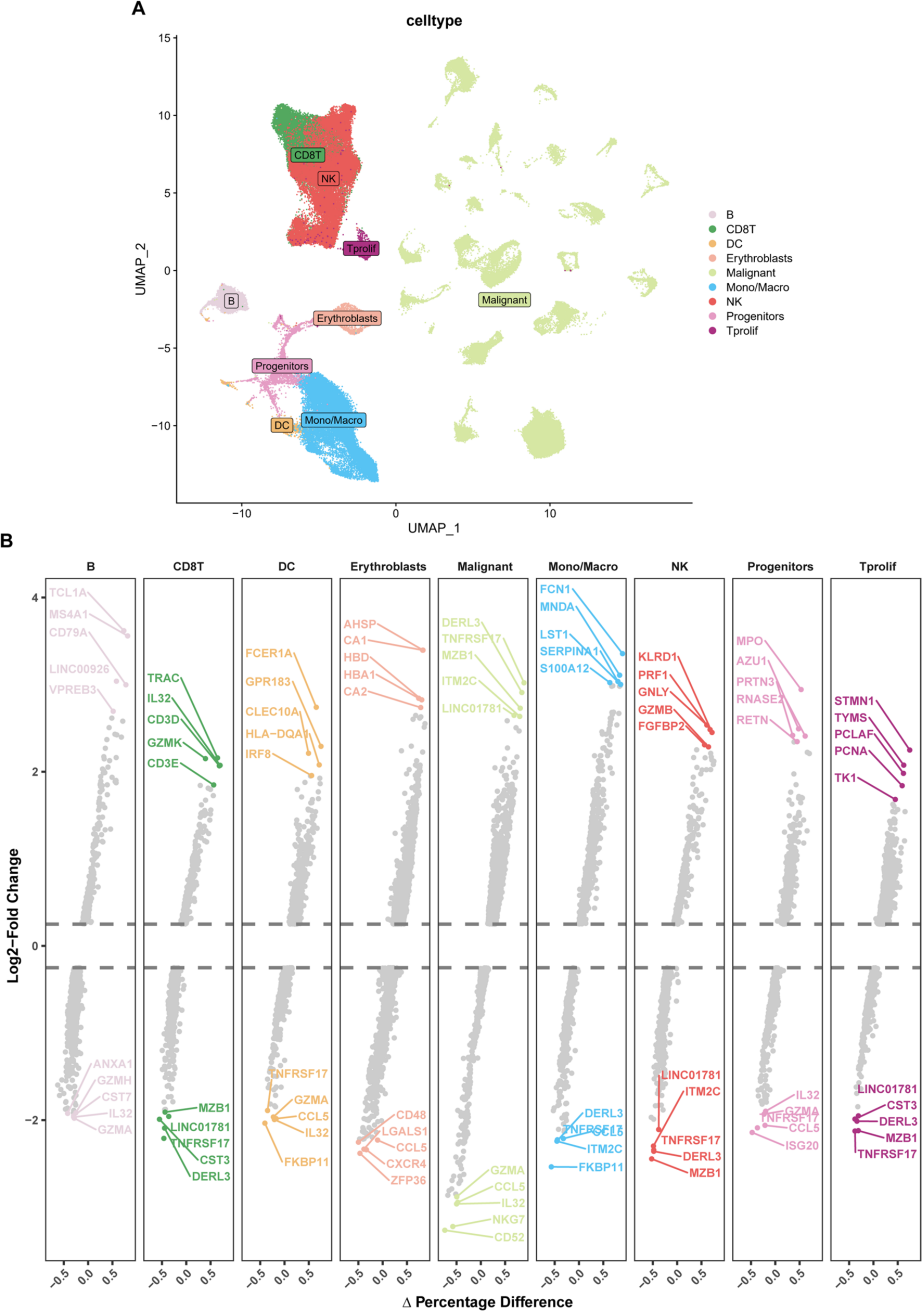
**A** UMAP plot, annotation of cellular subpopulations; **B** The top 5 high and low expression genes in each cell cluster within each cell cluster

In this investigation, we accessed the scRNA-seq dataset (GSE161801) in the TISCH database to obtain pretreatment data from 19 RRMM patients. Through the UMAP algorithm, followed by the annotation of different cell clusters, nine distinct cell clusters were identified: B, CD8 + T, DC, erythroblast, malignant, monocyte/macrophage, NK, progenitor, and Tprolif cell clusters. The UMAP visual representation (Fig. 1A) revealed that malignant cells constituted a significantly large cluster. Furthermore, the pronounced difference between groups of malignant cells indicates the presence of marked heterogeneity among malignant plasma cells (PCs).

**Figure 2:**
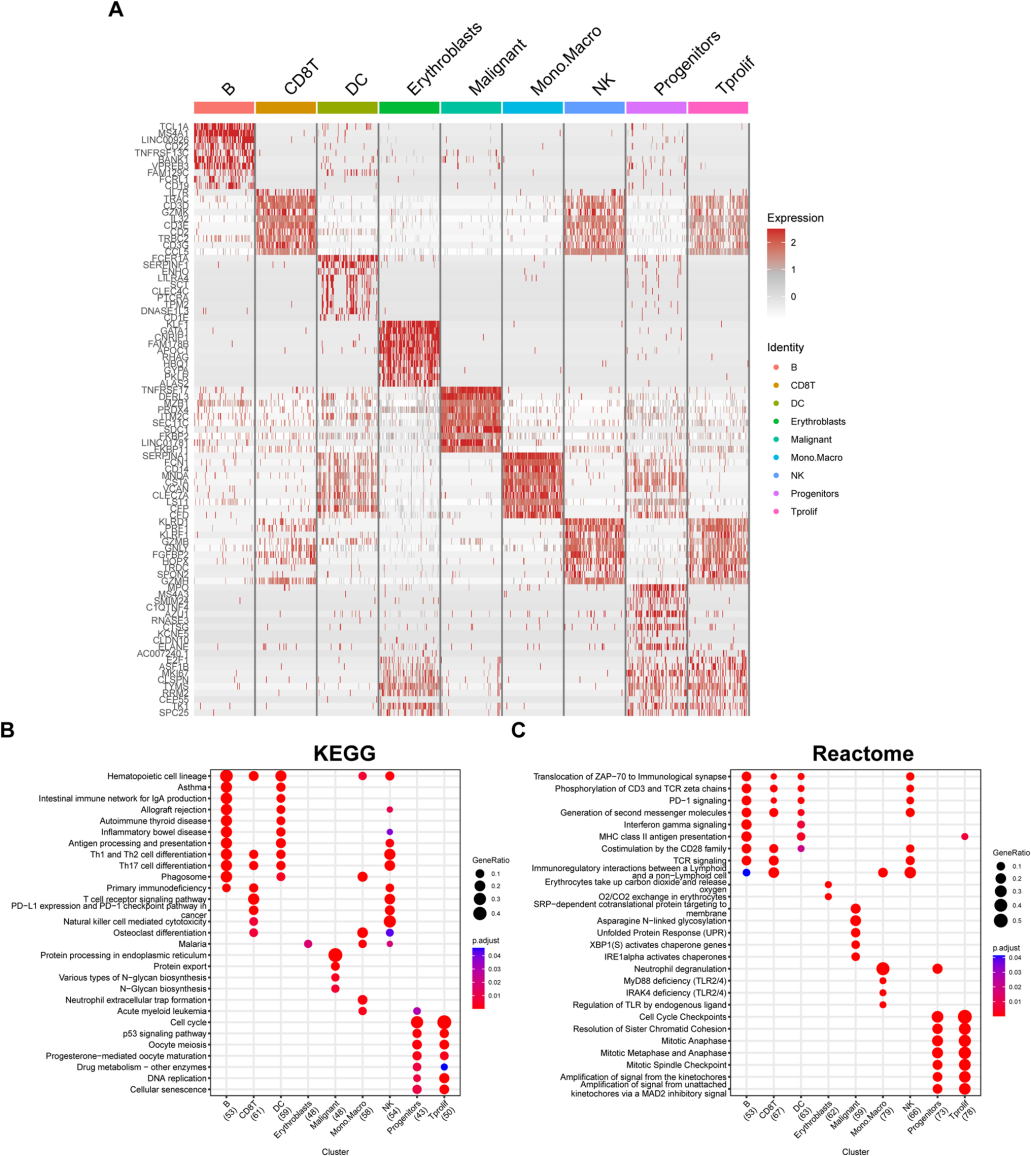
**A** Heatmap showing the top 10 marker genes in each cell cluster; **B** KEGG pathway enrichment of the top 100 marker genes in each cell cluster; **C** Reactome pathway enrichment of the top 100 marker genes in each cell cluster

The "FindAllMarkers" function in the R "Seurat" was used to identify the top five highly and lowly expressed genes in each cell cluster (Fig. 1B). Additionally, R "COSG" was utilized for the identification of marker genes for each cell type, displaying the top 10 highly expressed differentially expressed genes (DEGs) via a heatmap (Fig. 2A). The top 100 DEGs identified by "COSG" were subjected to enrichment analysis for KEGG and Reactome pathways, revealing that DEGs in B and DC cells shared similar enrichment pathways. Similarly, DEGs in CD8 + T and NK cells, along with Tprolif and progenitor cells, exhibited comparable enrichment pathways (Fig. 2B, 2C). KEGG pathway enrichment analysis of malignant PCs highlighted significant enrichment in processes such as protein processing in the endoplasmic reticulum, protein export, various types of N-glycan biosynthesis, and N-glycan biosynthesis. Reactome pathway enrichment analysis further indicated significant enrichment in asparagine N-linked glycosylation, SRP-dependent cotranslational protein targeting to membrane, unfolded protein response, xbp1s chaperone genes, and IRE1alpha-activated chaperones.

**Figure 3:**
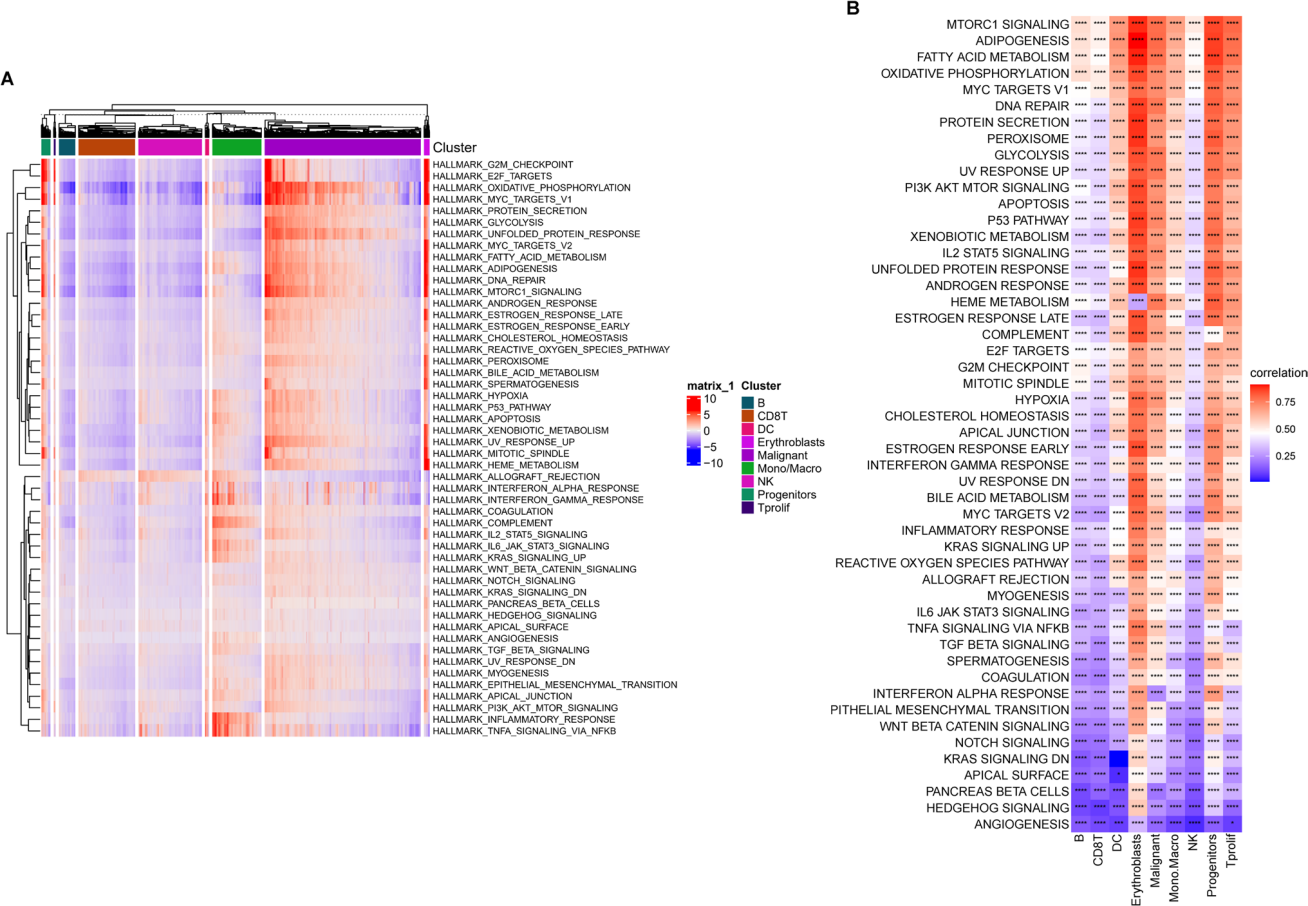
**A** Heatmap showing the hallmark pathway enrichment score of each cell; **B** Heatmap showing the correlation between LMES and each hallmark pathway

Subsequently, we identified 50 hallmark pathways from the MsigDB database. Using the R "ssGSEA," the lipid metabolism enrichment scores (LMESs) and the enrichment score of each hallmark pathway for each cell were calculated. Analysis revealed that erythroblasts, malignant cells, DCs, progenitors, and Tprolif cells exhibited increased enrichment scores in most hallmark pathways. Notably, malignant cells showed elevated hallmark pathway enrichment scores predominantly in OXIDATIVE PHOSPHORYLATION, MYC TARGETS V1, UNFOLDED PROTEIN RESPONSE, MTORC1 SIGNALING, ADIPOGENESIS, and DNA REPAIR (Fig. 3A). The LMES for each cell were calculated by employing R "GSVA," depending on the gene expression of LMRGs, and the correlation between LMES and the 50 hallmark pathways was analyzed. The results demonstrated a strong correlation between LMES and hallmark pathways in the erythroblast, malignant, progenitor, and Tprolif cell clusters, specifically in pathways such as the MTORC1 SIGNALING, ADIPOGENESIS, FATTY ACID METABOLISM, OXIDATIVE PHOSPHORYLATION, MYC TARGETS V1, PI3K AKT MTOR SIGNALING, APOPTOSIS, P53 PATHWAY, DNA REPAIR, and GLYCOLYSIS pathways. However, LMES exhibited a weak correlation with most hallmark pathways in CD8 + T, NK, and B cells (Fig. 3B).

**Figure 4:**
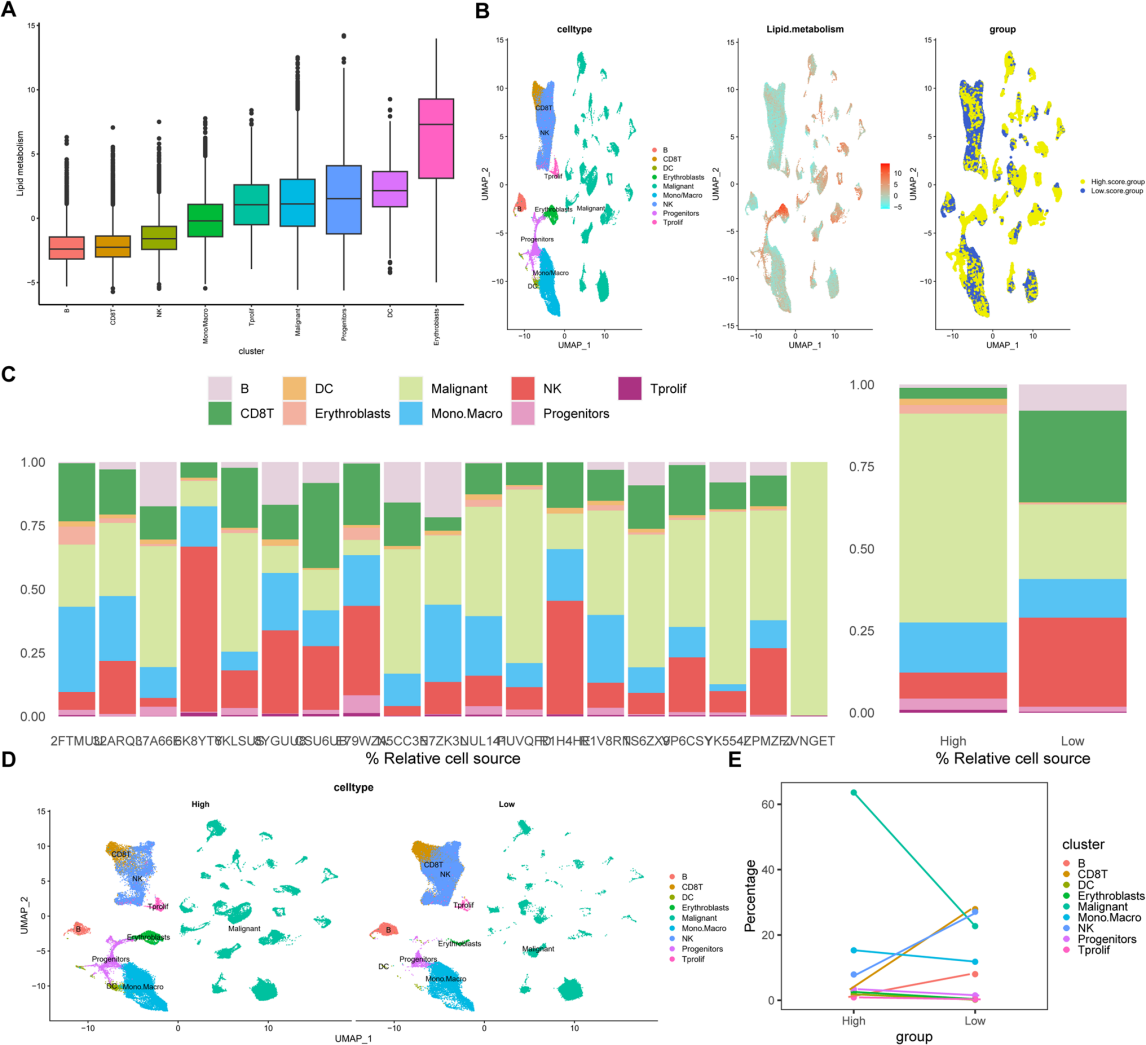
**A** The distribution of the LMESs in different cell types; **B** UMAP showing the distribution of LMESs in different cell types and the distribution of high and low score groups in different cell types; **C** Relative percentages of 9 cell clusters in different sample sources, and the percentages of different cell types in high and low LMES groups; **D** UMAP shows the distribution of various cell types in high and low LMES groups; **E **The trend changes in the proportion of various cells in high and low LMES groups

The LMES values for each cell type are displayed in Fig. 4A, and a visual UMAP indicating the distribution of LMES across each cell cluster was created (Fig. 4B), along with the percentage of various cells in each sample (Fig. 4C). The LMES values of the cell clusters, from highest to lowest, were as follows: erythroblasts, DCs, progenitors, malignant cells, Tprolif cells, monocytes/macrophages, NK cells, CD8 + T cells, and B cells. The percentages of B (1% vs. 8%), CD8 + T (3.3% vs. 27.9%), and NK cells (7.9% vs. 27.1%) were significantly lower in the high LMES group. Conversely, the percentages of malignant (63.6% vs. 22.6%), monocyte/macrophage (15.3% vs. 11.8%), erythroblast (2.6% vs. 0.4%), progenitor (3.4% vs. 1.5%), Tprolif (0.9% vs. 0.3%), and DC cells (1.9% vs. 0.2%) were significantly greater (Fig. 4C–E). These findings suggest that lipid metabolism may influence immune cell infiltration.

## Construction and evaluation of the LMRPgenes predictive model based on multiple machine learning methods

**Figure 5:**
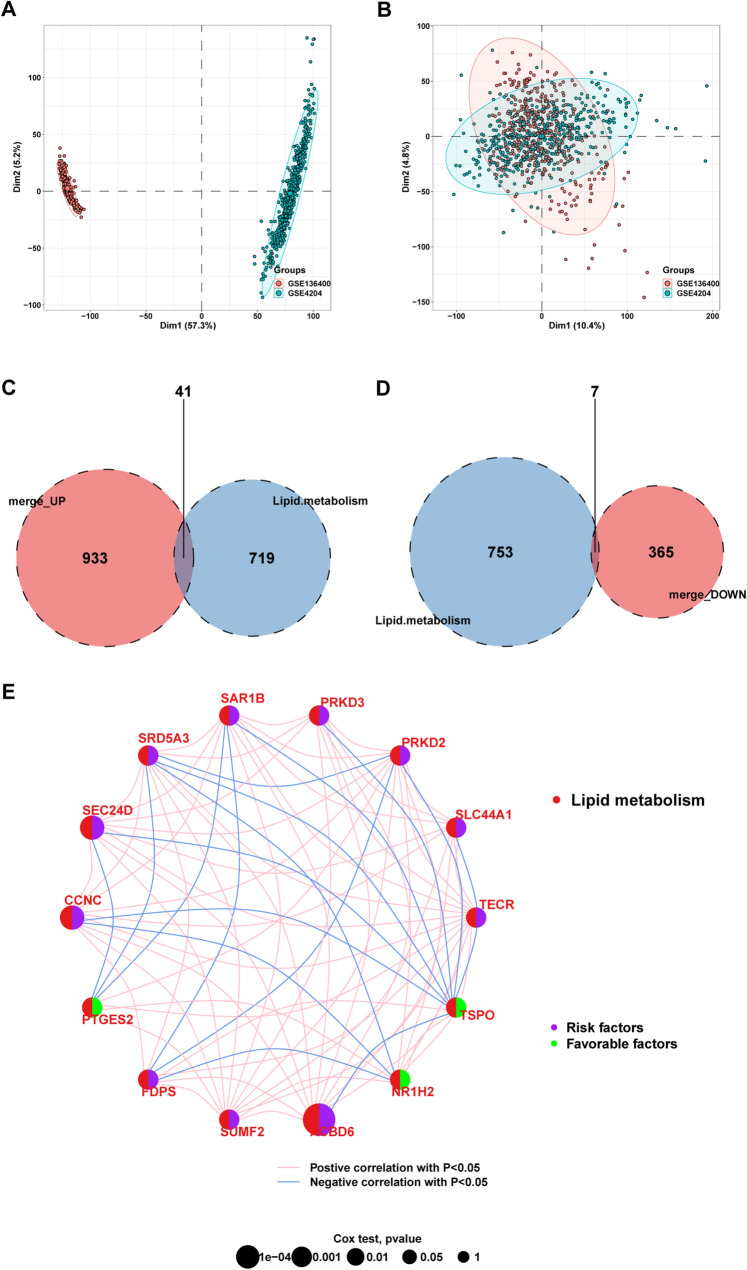
**A**, **B** PCA before and after batch effect removal; **C**, **D** Venn plot showing the intersecting genes between up/downregulated DEGs and LMRGs; **E **Results of univariate Cox regression analysis of LMRPgenes and the correlations between these genes. The circle size represents the *p* value of the univariate Cox regression analysis

In this study, the GSE4204 (559 pretreatment samples) and GSE136400 (354 pretreatment samples) cohorts were obtained from the GEO database. The R packages “limma” and “sva” were used to eliminate batch effects in the two datasets. Following this process, a merged dataset containing 892 samples and 16,729 genes was compiled. The results of principal component analysis (PCA) before and after batch effect removal are displayed in Fig. 5A, B.

**Figure 6:**
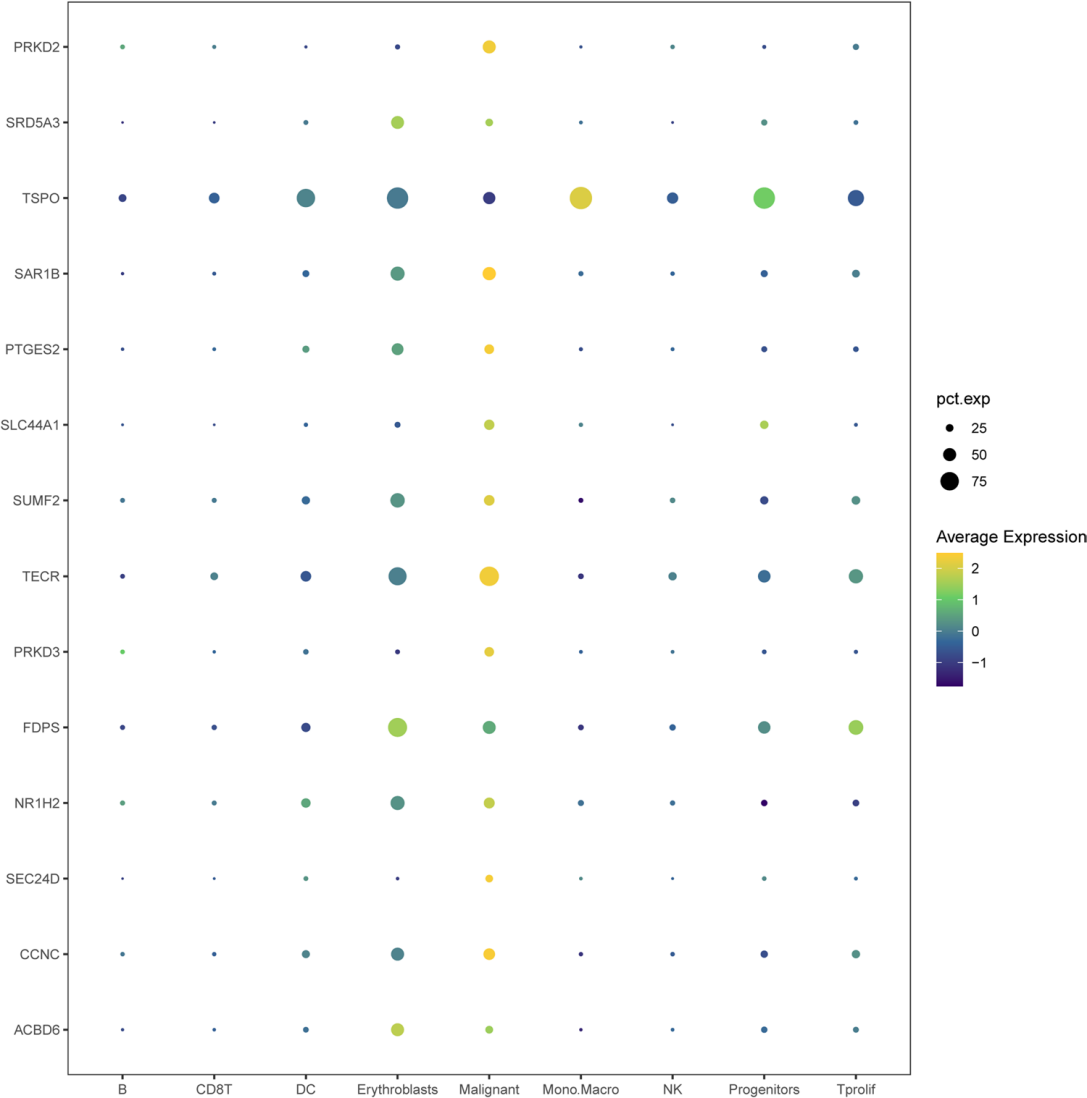
The expression of 14 LMRPgenes in single-cell sequencing data

The analysis identified 41 upregulated and 7 downregulated LMR-DEGs from an initial set of 1346 DEGs intersecting with 760 LMRGs (Fig. 5C, D). Further analyses, including correlation analysis, univariate Cox regression, and K‒M survival analysis, led to the identification of 14 LMRPgenes: ACBD6, CCNC, SEC24D, NR1H2, FDPS, PRKD3, TECR, SUMF2, SLC44A1, PTGES2, SAR1B, TSPO, SRD5A3, and PRKD2. TSPO, NR1H2, and PTGES2 were found to act as protective factors, whereas the remaining factors were considered risk factors (Fig. 5E). Additionally, the single-cell expression of these 14 LMRPgenes is depicted in a bubble chart (Fig. 6).

**Figure 7:**
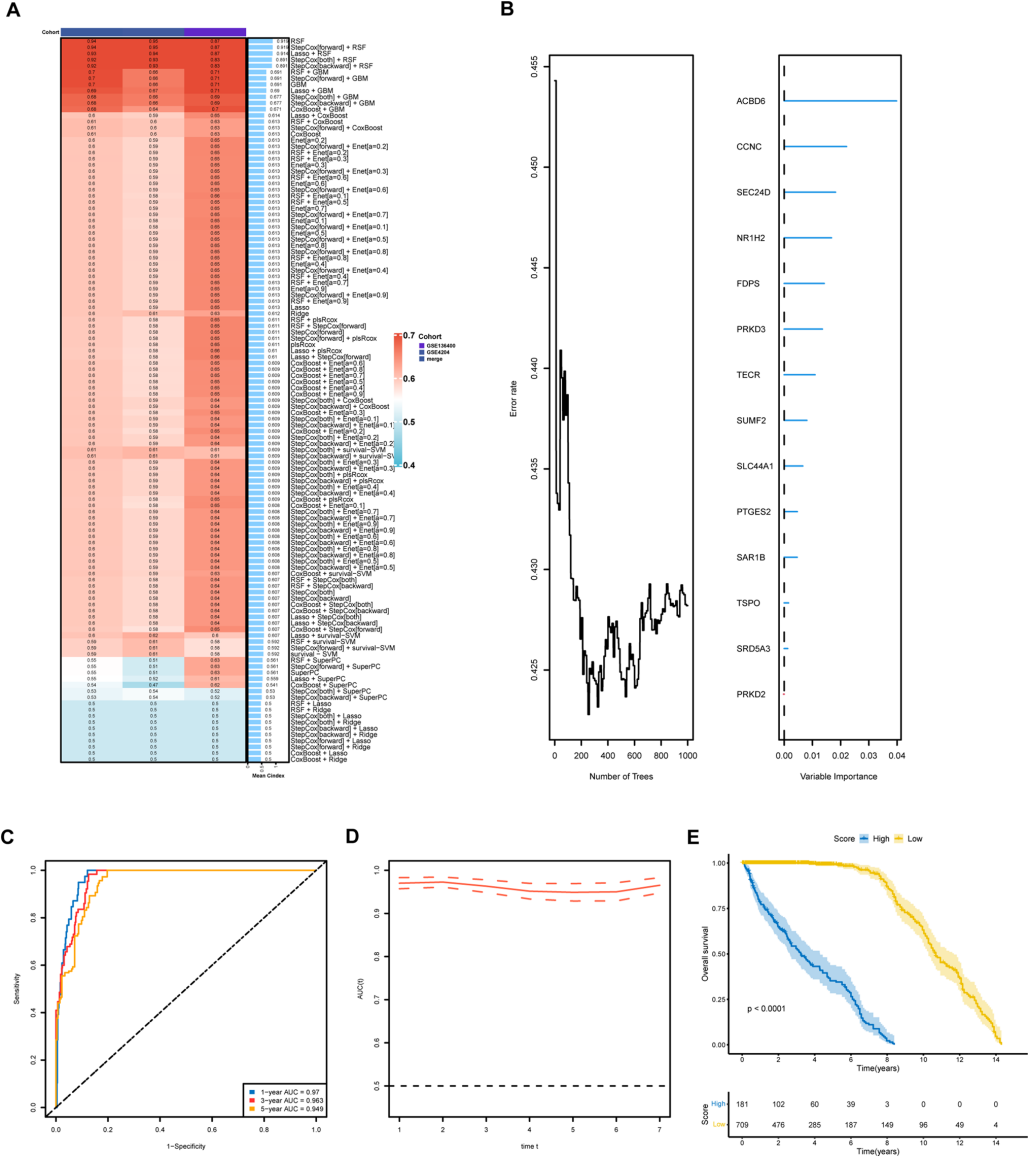
**A** Construction of multiple machine learning models; **B** Prediction model based on random forest algorithm and variable importance of LMRPgenes; **C** ROC curves of the training cohort; **D** Time-dependent ROC curve of the training cohort; **E** Kaplan–Meier curves of RS in the training cohort

Predictive models based on the 14 LMRPgenes were created using ten machine learning algorithms and 117 combinations. The merged dataset served as the training set, and the GSE4204 and GSE136400 cohorts were used to evaluate the predictive power separately. The concordance index (C-index) and the average C-index for the three datasets were calculated for each algorithm combination to evaluate their predictive capabilities. The RSF-based model achieved an average C-index of 0.919, with a value of 0.94 in the training set, 0.95 for GSE4204, and 0.87 for GSE136400 (Fig. 7A). Consequently, RSF was employed to construct the LMRPgenes prediction model for MM. Based on RSF variable importance, the genes were ranked in descending order of significance (Fig. 7B).

**Figure 8:**
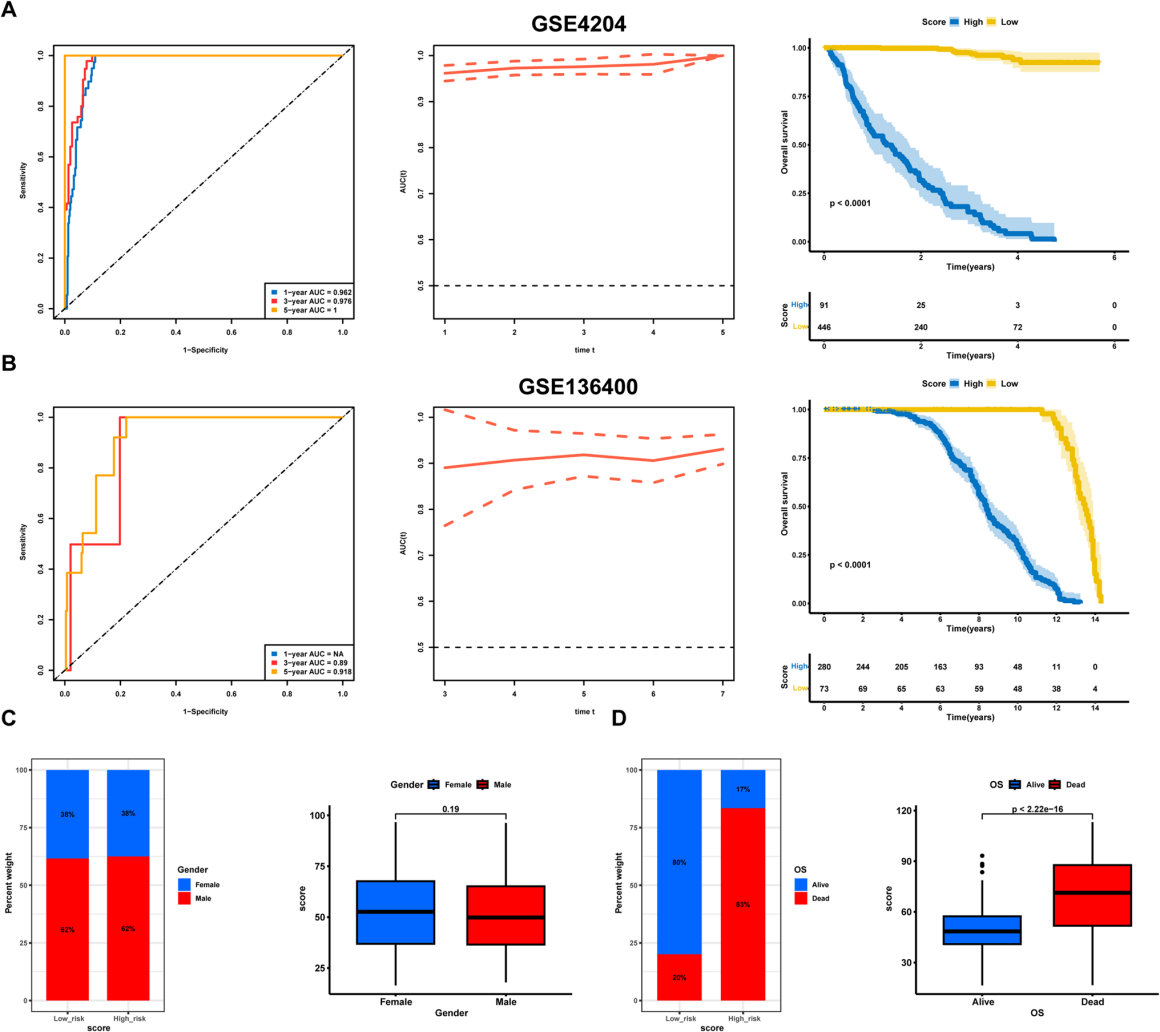
**A**, **B** ROC curves, time-dependent ROC curve, and Kaplan–Meier curves of RS for the cohort GSE4204 and GSE136400; **C** Relationship between model RS and gender; 8D. Relationship between model RS and OS

The effectiveness of the model's predictions was evaluated by ROC curve analysis. For the training set, the AUC values of the 1-, 3-, and 5-year intervals were 0.97, 0.963, and 0.949, respectively (Fig. 7C). The area under the curve (AUC) of the time-dependent ROC curve exceeded 0.9 (Fig. 7D). For the GSE4204 dataset, the 1-, 3-, and 5-year AUC values were 0.962, 0.976, and 1.0, respectively, with the AUC values approaching 1 over time (Fig. 8A). In GSE136400, the 3- and 5-year AUC values were 0.89 and 0.918, respectively, with the AUC values exceeding 0.75 and increasing over time (Fig. 8B). These results underscore the model’s excellent predictive validity across the three datasets.

Survival status changes were evaluated in these three datasets for different RS groups. The results showed that patients with a high RS exhibited significantly lower OS than those with a low RS (Figs. 7E, 8A, B). Moreover, the relationships between the RS, gender, and survival status were evaluated, revealing no significant gender difference between the two groups (Fig. 8C). However, a significant increase in mortality rate was observed in the high-RS group compared to the low-RS group (83% vs. 20%), with a notable difference in RS values between the alive and dead groups (*p* < 2.22e−16) (Fig. 8D).

**Figure 9:**
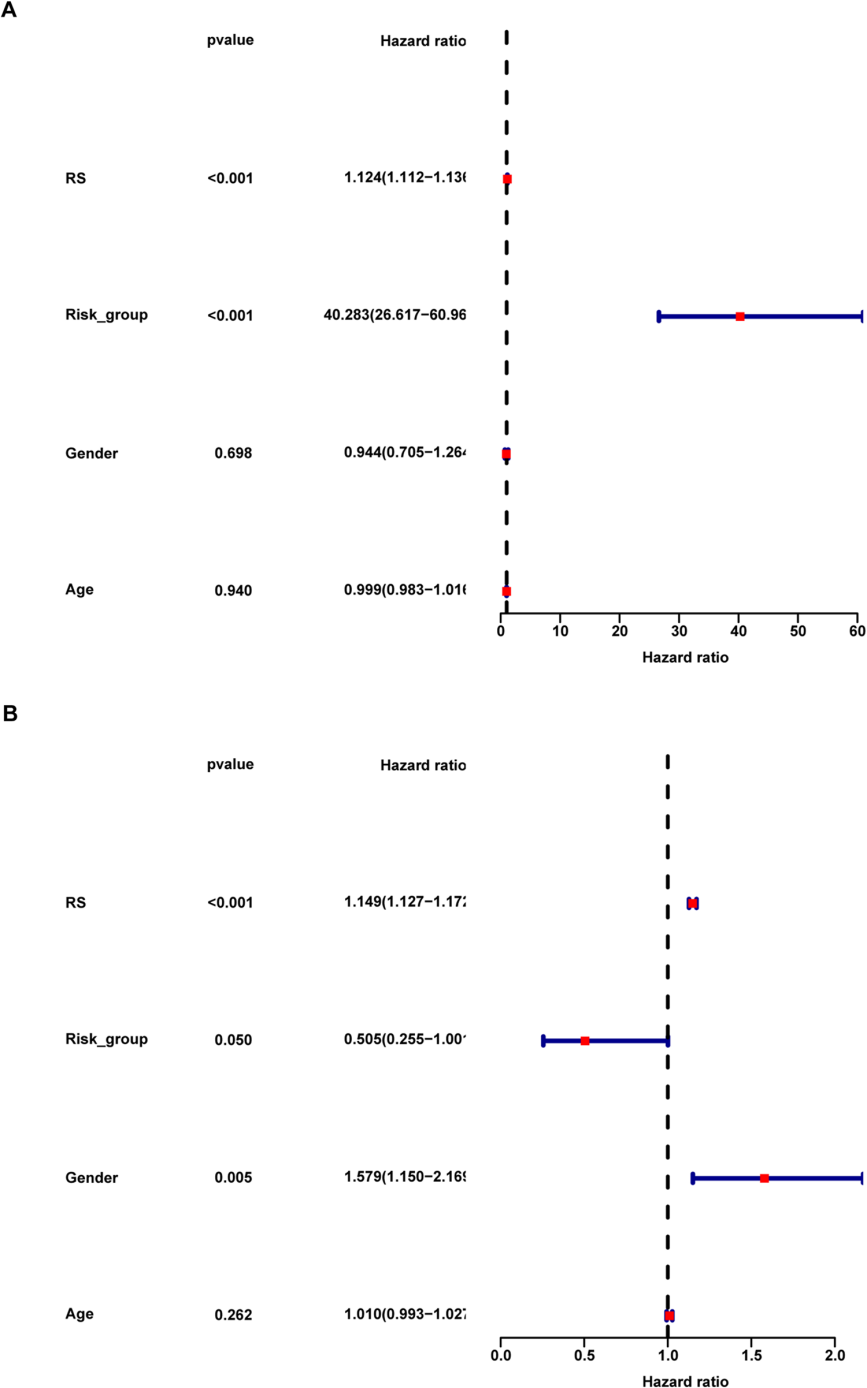
**A**, **B** Univariate and multivariate analyses of the clinical characteristics, RS_group, and RS for the OS

Subsequently, we conducted univariate (Fig. 9A) and multivariate (Fig. 9B) Cox regression analyses to determine whether the RS is an independent prognostic factor for predicting the survival of MM patients, along with gender, age, and RS group. The findings indicated that the RS (HR: 1.149, CI 1.127–1.172, *p* < 0.001) served as a potent prognostic predictor, indicating the potential ability to predict the outcomes of MM patients.

## Enrichment analysis of RS-related genes

**Figure 10:**
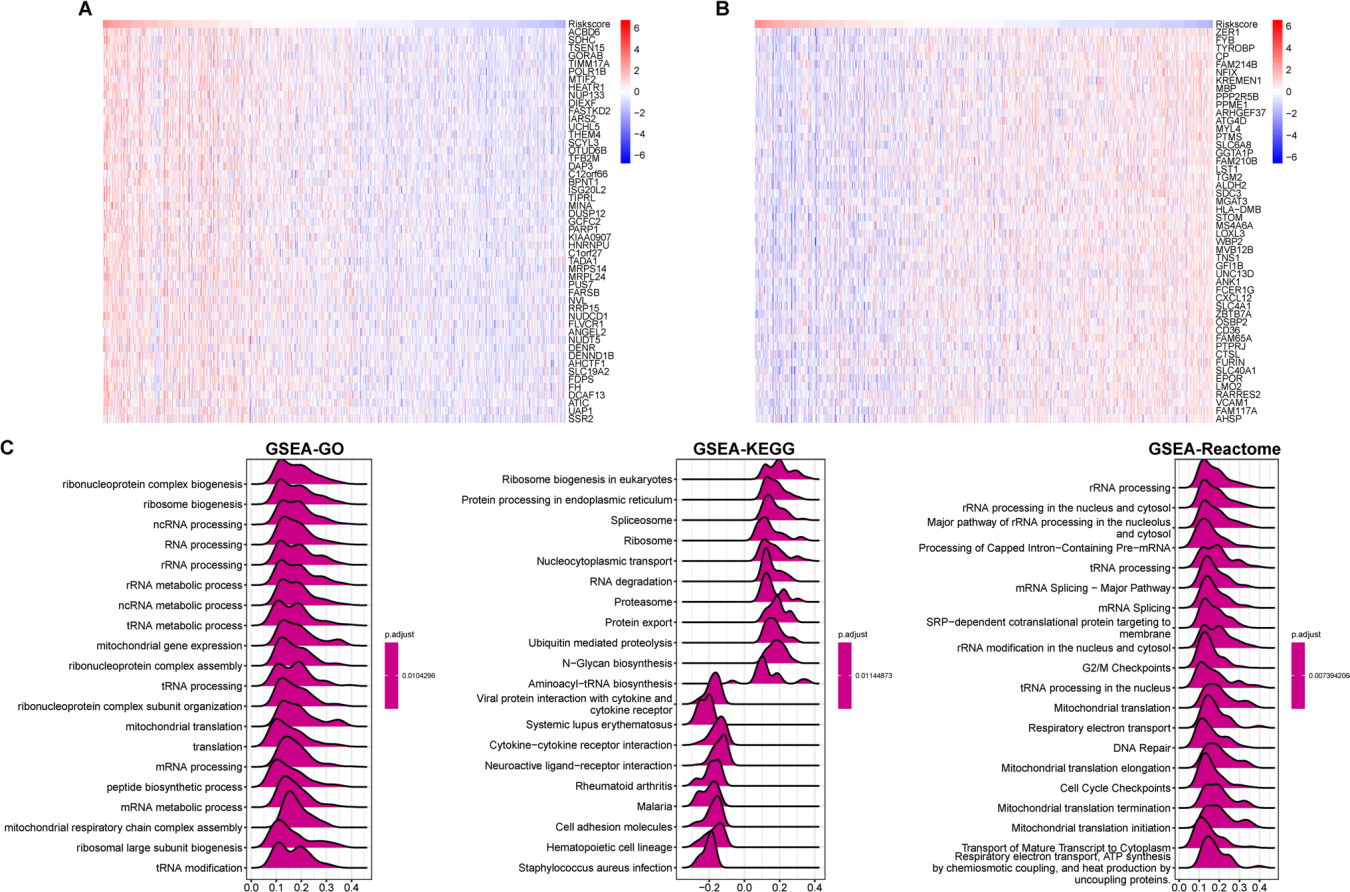
**A**, **B** Heatmap showing the expression of the top 50 genes positively and negatively related to RS; **C** GSEA enrichment analysis on genes positively and negatively related to RS, including GO KEGG and Reactome pathways

We conducted a correlation analysis between RS and all genes, identifying the top 50 genes that were positively or negatively correlated with RS, as depicted in a heatmap (Fig. 10A, B). Following the correlation analysis, GSEA, including GO, KEGG, and Reactome pathway analyses, was performed (Fig. 10C). This analysis revealed that genes closely associated with RS were predominantly enriched in three key areas:

- 3.1 The biogenesis of ribosomes and ribonucleoprotein complexes, the biogenesis of the ribosomal large subunit, the processing and metabolism of various RNAs, and the translation, modification, and translocation of proteins.
- 3.2 Expression of mitochondrial genes, mitochondrial translation, the composition of the respiratory chain complex, and ATP biosynthesis.
- 3.3 Interactions between cytokines and receptors and disorders related to autoimmunity and infections.

KEGG analysis revealed a normalized enrichment score (NES) < -2 (*p* value < 0.05; FDR < 0.25) for pathways involving viral protein interactions with cytokine receptors, systemic lupus erythematosus, cytokine-receptor interactions, neuroactive ligand‒receptor interactions, rheumatoid arthritis, malaria, cell adhesion molecules, and Staphylococcus infection. This finding suggested that the expression of RS-related genes expression was downregulated in several inflammation-related pathways.

## Immunological analysis

**Figure 11:**
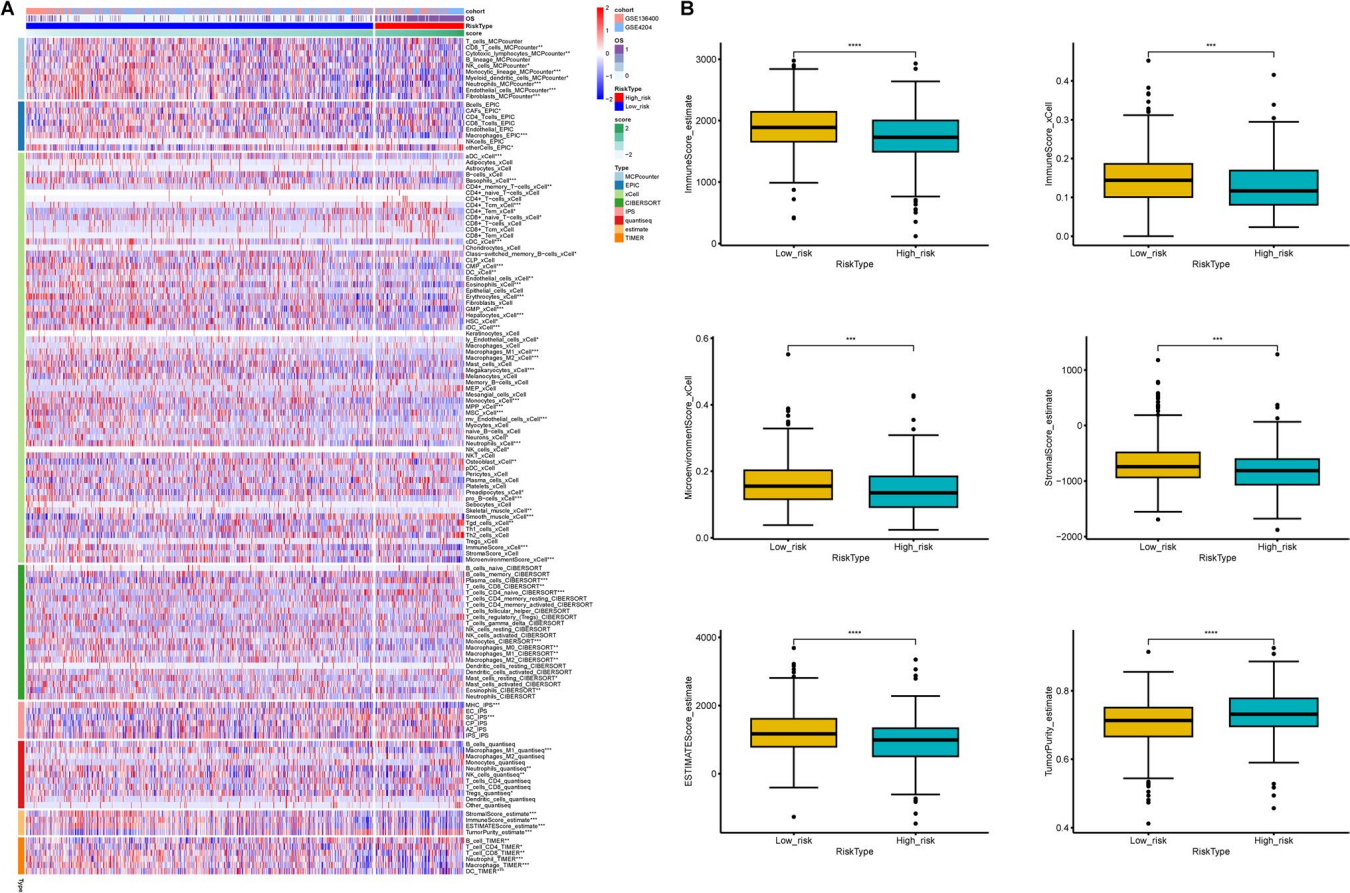
**A** Heatmap showing infiltration of immune cells in high- and low-RS groups based on MCPcounter, EPIC, xCell, CIBERSORT, IPS, quanTIseq, ESTIMATE, and TIMER algorithms; **B** The relationship between the model RS and the immune score related to the microenvironment based on ESTIMATE and IPS algorithms

We employed the R “IOBR” for analyzing the immune status of the BMM in MM patients using eight algorithms: MCPcounter, EPIC, xCell, CIBERSORT, IPS, quanTIseq, ESTIMATE, and TIMER. ESTIMATE was used to assess the infiltration levels of immune cells within MM samples. The group with a higher RS had significantly lower stromal, immune, and ESTIMATE scores but notably higher tumor purity scores (Fig. 11B). Additionally, through the immunophenotyping score (IPS), we separately calculated the scores for four distinct immunophenotypes, revealing a negative correlation between the RS and the major histocompatibility complex (MHC) score and a positive correlation with the suppressor cell (SC) score. These findings suggest that a reduction in antigen presentation and an increase in suppressive immunity may facilitate tumor cell immune evasion, thereby diminishing immune-mediated tumor cell clearance and further promoting tumorigenesis.

Using the MCPcounter algorithm, we quantified the infiltration of ten types of immune cells in MM samples. The results revealed that the high-RS group exhibited significant decreases in the infiltration of six immune cell types and two stromal cell types, namely, neutrophils, monocytes, fibroblasts, cytotoxic lymphocytes, endothelial cells, CD8 + T cells, NK cells, and myeloid dendritic cells. CIBERSORT was also utilized to assess the infiltration levels of 22 immune cell types, revealing diminished infiltration of CD8 + T cells, monocytes, macrophages (M1, M2, and M0 types), and eosinophils, except for CD4 + naive and plasma cells. Verifying these results through additional algorithms yielded similar findings, collectively suggesting a decrease in the infiltration of most immune cells within the high-RS group (Fig. 11A).

**Figure 12:**
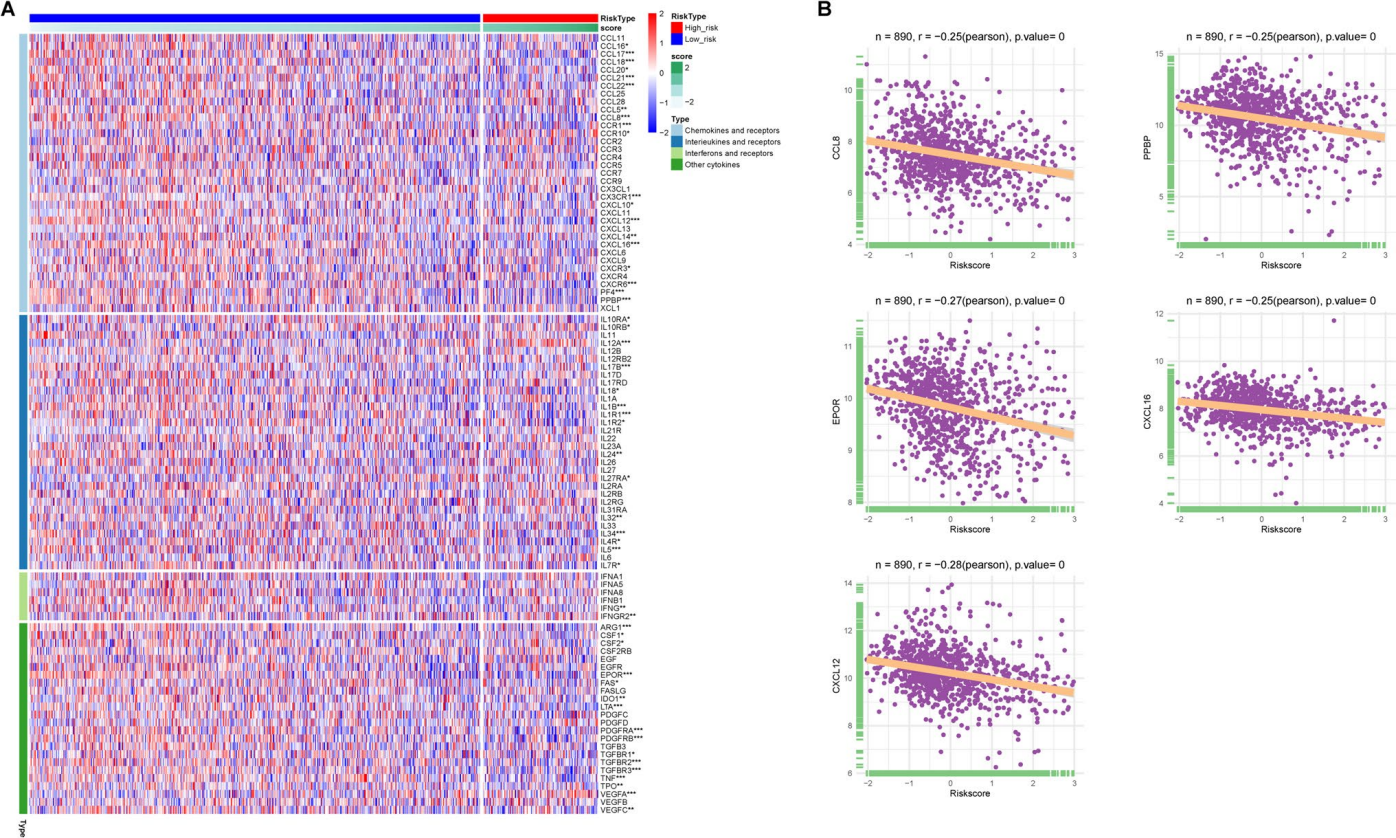
**A**, **B** Heatmap showing the relationship between the model RS and cytokines and their receptors; (showing *r* < −0.25)

This analysis of immune status within the MM dataset indicated a substantial reduction in immune cell infiltration in the high-RS group. Moreover, by correlation analysis, we evaluated the relationships between the RS and the expression of various cytokines (including chemokines, interleukins, and interferons) and their receptors (Fig. 12A). The analysis revealed a predominantly negative correlation between RS and most cytokines, notably CCL8, PPBP, CXCL16, EPOR, and CXCL12 (Fig. 12B). The above results showed that BMMs with a high RS are likely to be in a state of immunosuppression, which could contribute to the adverse prognosis observed in this group.

**Figure 13:**
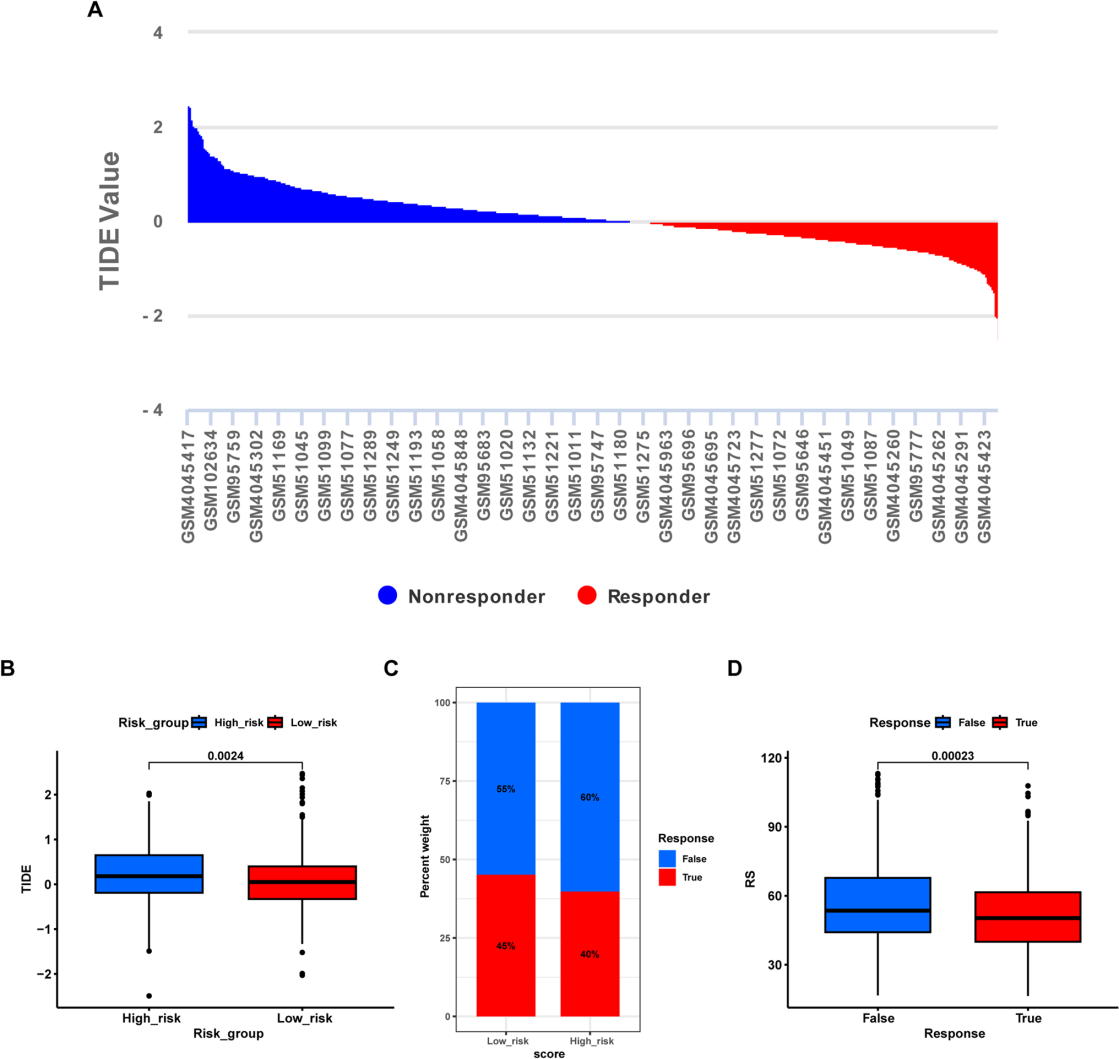
**A** The TIDE analysis reveals a positive correlation between the TIDE score and RS TIDE (the higher RS, the worse the immunotherapy effect); **B** Box plot showing the TIDE scores difference in high- and low-RS groups; **C** Percentage of immunotherapy response in high- and low-RS groups; **D** Difference of model RS in different immunotherapy response groups

Subsequently, we investigated whether the immunosuppression status of MM patients in the high-RS group affects the effectiveness of immunotherapy. The TIDE database was used to predict the relationship between immunotherapy efficacy and the model RS. TIDE analysis indicated that the TIDE score was positively related to the RS (Fig. 13A), and the high-RS group had a significantly greater TIDE score than did the low-RS group (*p* value: 0.0024) (Fig. 13B), suggesting diminished efficacy of immunotherapy. Moreover, there was a weaker positive immunotherapy response in the high-RS group than in the low-RS group (40% vs. 45%) (Fig. 13C), and a significant decrease in the RS was noted in the group responding to immunotherapy (*p* value: 0.00023) (Fig. 13D).

## Prediction of drug sensitivity to nonimmunotherapeutic treatments

**Figure 14:**
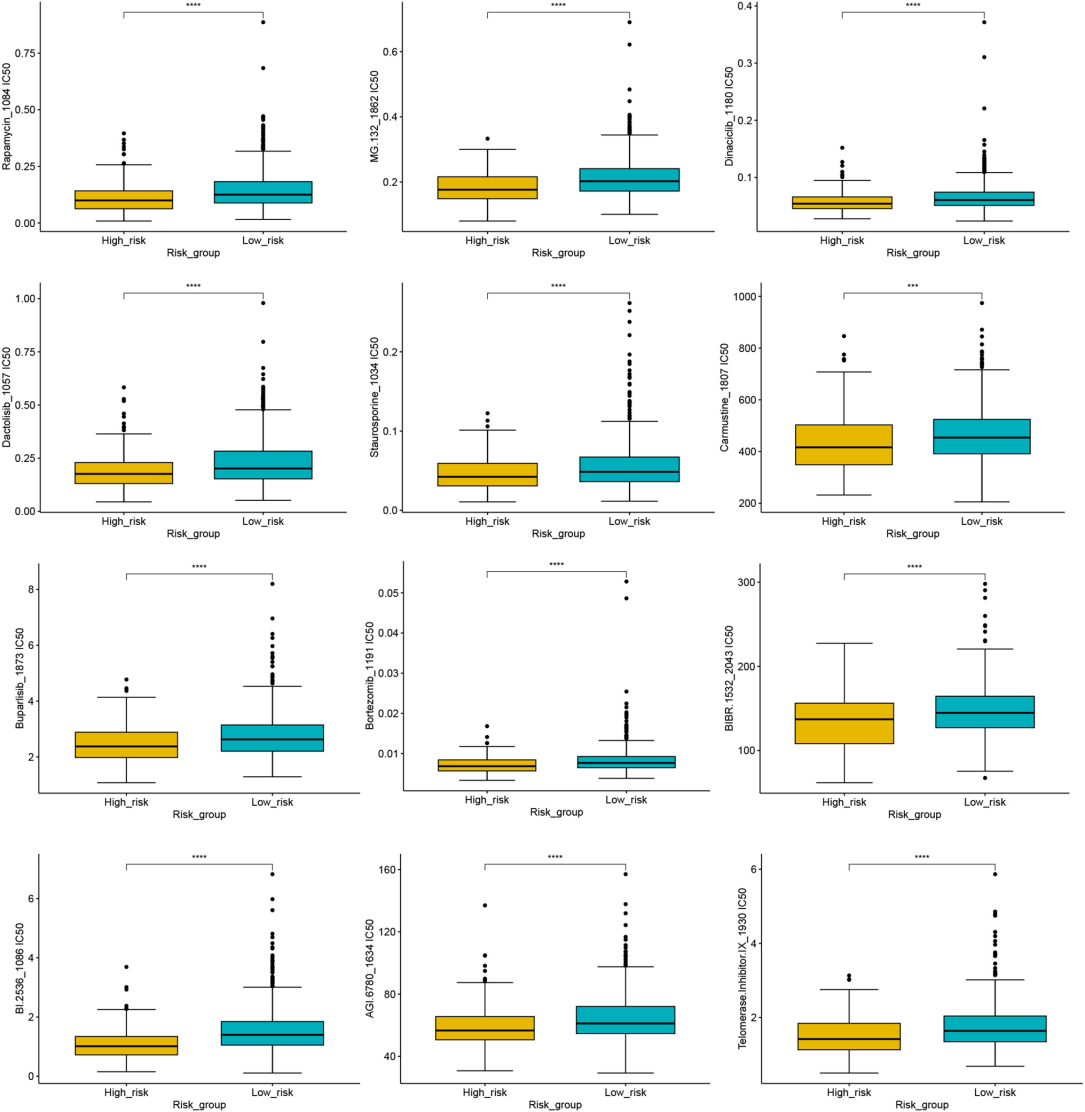
The IC50 values of different anticancer drugs in high- and low-RS groups based on R package “oncoPredict.”

Our study revealed that patients with a high RS are characterized by an immunosuppressive microenvironment and exhibit diminished responses to immunotherapy. To identify more effective treatment options for patients with a high RS, we used the R package “OncoPredict” to determine the IC50 values of various anticancer drugs. The IC50 value serves as an indicator of treatment sensitivity, wherein higher IC50 values signify reduced sensitivity. A comparative analysis was conducted to evaluate the differences in the IC50 values of various anticancer drugs between the high- and low-RS cohorts. The classical proteasome inhibitor bortezomib demonstrated notable therapeutic efficacy in the high-RS group. Additionally, other anticancer drugs, including the panclass I PI3K inhibitor buparlisib, the multicell cycle protein-dependent kinase inhibitor dinaciclib, staurosporine, the proteasome inhibitor MG.132, rapamycin, the telomerase inhibitor MST-312, and the heat shock protein 90 inhibitor luminespib, were observed to have lower IC50 values, indicating significant differences in efficacy between the two RS groups (Fig. 14). These findings suggest that these drugs, either individually or in combination, may offer novel therapeutic avenues for MM patients characterized by high-RS and suboptimal immunotherapy outcomes.

## Results of RT-PCR

**Figure 15:**
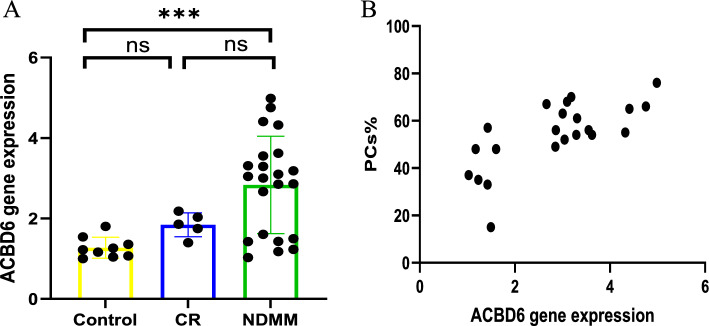
**A** ACBD6 expression levels among different groups; **B** Correlation analysis between ACBD6 gene expression and the percentage of malignant PCs

Our analysis focused on the expression levels of the ACBD6 gene across NDMM, CR, and control groups. Notably, ACBD6 was significantly upregulated in NDMM group compared to control group (*p* = 0.001). No significant differences in ACBD6 expression were observed when comparing the CR and control groups, as well as the NDMM and CR groups (Fig. 15). Additionally, the gene expression level of ACBD6 was significantly positive correlated with the percentage of malignant PCs in bone marrow(r: 0.7163; *p*: 0.0002).

## Discussion

Arising from stromal stem cells, BM adipose tissue (BMAT) plays a pivotal role in the progression of multiple myeloma (MM). Two categories of BMAT have been identified based on their anatomic locations: proximal-regulated rBMAT and distal-constitutive cBMAT. The latter tends to aggregate in areas with lower skeletal activity, potentially negatively impacting hematopoiesis. In contrast, rBMAT is intertwined with hematopoietic cells surrounding growth plates and bone trabeculae [18–21]. BM adipocytes not only store energy but also release FFAs and produce a range of signaling molecules like leptin, adiponectin, IL-6, TNFα, and IGF-1. These molecules promote myeloma cell proliferation, migration, and resistance to chemotherapy [20, 22]. BM adipocytes from MM patients have been shown to foster myeloma growth and shield these cells from the effects of chemotherapy-induced cell apoptosis [23, 24].

Panaroni C et al. have revealed that malignant PCs from MM patients trigger lipolysis in BM adipocytes, enhancing gene expression linked to lipolysis during different stages of the disease [13]. It has been demonstrated that etomoxir inhibits fatty acid oxidation(FAO) by blocking CPT1, the rate-limiting enzyme for FAO, thereby decreasing intracellular ATP levels and leading to cell cycle arrest in the G0/G1 phase of MM cells [25]. In addition, inhibiting FA synthesis has shown promise in combating tumor growth. A lncRNA derived from the MIR17HG, lnc-17–92, has been found to accelerate tumor growth by upregulating ACACA (an ACC1 encoding gene). Disrupting lnc-17–92 with an antisense oligonucleotide yielded significant tumor suppression in vitro and in vivo [26]. Overall, the dynamics of FA metabolism—both its synthesis and breakdown—are crucial in the progression, manifestation, and treatment resistance of multiple myeloma.

Increasing evidence suggests that BM stromal cells shield tumor stem cells while fostering an immunosuppressive microenvironment, hindering the eradication of minimal residual disease (MRD) and fostering resistance. CD8 + T cells, pivotal to the adaptive immune response, play a crucial role in anti-tumor immunity. Recent findings revealed a marked reduction in both the quantity and functionality of CD8 + T in BM of MM patients. Moreover, BM CD8 + T function lagged behind that of matched peripheral circulation. In vitro assays unveiled that compromised BM CD8 + T function and diminished mitochondrial mass in MM patients correlated with heightened long-chain FAs uptake, primarily driven by elevated FATP1 expression, with FATP1 inhibition ameliorating CD8 + T cell dysfunction [27]. Furthermore, CD36 facilitated CD8 + T lipid peroxidation and ferroptosis through the absorption of FAs enriched in BMM of MM, culminating in CD8 + T effector function impairment, while arachidonic acid (AA) emerged as the principal FA in BMM, triggering ferroptosis in CD8 + T [28]. Collectively, these studies underscore the impact of aberrant lipid metabolism within the BMM on CD8 + T abundance and functionality. Our analysis of the RRMM single-cell dataset showed a significant decrease in CD8 + T abundance in the high LEMS group compared to the low LEMS group. Moreover, CD8 + T infiltration decreased with the RS of the predictive model elevated. Hence, dysregulated lipid metabolism may influence CD8 + T cell infiltration; however, the precise effects require further experimental validation.

Chimeric antigen receptor (CAR) T cell therapy stands out as a novel and promising targeted approach that has demonstrated satisfactory efficacy in patients with RRMM in recent years. However, the occurrence of cytokine release syndrome (CRS) and the clinical outcomes present significant barriers, hampering the widespread clinical application of CAR-T. Platelet-activating factor (PAF), also known as 1-alkyl-2-acetyl-sn-glycero-3-phosphocholine, has garnered attention [29]. A recent investigation unveiled a distinctive metabolic profile in RRMM patients experiencing CRS and exhibiting poor therapeutic responses. The study observed a significant reduction in the PAF-like molecule intermediate glycerophosphocholine (GPC) alongside a notable elevation in lysophosphatidylcholine (LPC) levels during CRS. This metabolic profile may hold crucial predictive value for the efficacy of CAR-T therapy in RRMM patients. The study suggests that CAR-T therapy may exert its effectiveness by inhibiting the remodeling of PAF synthesis through LPCAT1 inactivation, consequently leading to increased LPC levels in plasma [30]. Targeting LPCAT1 and PAF remodeling emerges as a promising avenue to bolster MM CAR-T therapy.

LPCAT1, a member of the LPCAT family, plays a pivotal role in LD formation and LPC metabolism. Remarkably, the study revealed a significant upregulation of LPCAT1 expression in MM patients, correlating with poorer OS, a finding validated across two separate cohorts of MM patients [30]. ACBD6 (Acyl-CoA binding domain containing 6)), a protein involved in regulating various lipid metabolic processes, was highlighted. ACBD6 facilitates acyl donors for LPCAT1, which catalyzes the acylation of monoacyl chain lipids like lysophosphatidic acid (LPA) and LPC. Deficiencies in LPCAT1 enzyme-mediated phosphatidylcholine formation from LPC lead to reduced LD content [31]. LDs, essential organelles in lipid metabolism, not only regulate intracellular cytoplasmic and lipid metabolism but also play vital roles in cell signaling, intracellular and extracellular transport, and gene transcriptional regulation [32]. Aberrations in LDs or fatty acids contribute to cancer progression and metastasis. Consequently, several enzymes involved in LD formation or breakdown, such as ACC, fatty acid synthase (FASN), 3-hydroxy-3-methylglutaryl monoacyl-CoA reductase (HMGCR), and stearoyl-CoA desaturase (SCD), have been identified as potential therapeutic targets in cancer treatment.

Moffitt Cancer Center revealed that acidic alterations in the tumor microenvironment, sensed by OGR1, promote LD formation. OGR1 activates downstream signaling through protein phospholipase C and PI3K/AKT, fostering LD formation from amino acid products and promoting tumor progression [33]. Xu et al. demonstrated that proteasome inhibitors (PIs) induce the accumulation of abnormal LDs in MM, predominantly triglycerides, via activation of the ATF4/SREBP pathway. This LD accumulation serves as a compensatory mechanism to enable protein synthesis, mitigate endoplasmic reticulum stress, and sustain cell survival, ultimately conferring resistance to PIs [34]. Thus, drugs targeting LD formation and catabolism offer promising avenues for MM therapy.

ACBD6 plays a crucial role as an acyl donor, specifically binding long-chain acyl-coenzyme A to regulate lipid synthesis, LD formation, and protein and lipid esterification [31, 35]. However, its role and mechanisms in MM remain underexplored. Our research leveraging both scRNA-seq and bulk-seq data revealed significant differences in ACBD6 expression between normal and tumor tissues in MM, with high ACBD6 expression correlating with notably lower OS (*p* < 0.001). Our analyses of BM samples from 22 NDMM patients, 5 patients in complete remission(CR), and 9 healthy controls indicated that ACBD6 levels were markedly higher in the MM group compared to the control group. Additionally, a strong correlation was found between ACBD6 expression levels and plasma cell counts in the BM for NDMM patients.

ACBD6 is not ubiquitously expressed but is found in specific cell types, such as blood CD34 + progenitor cells and perivascular CD31 + endothelial cells, contributing to hematopoiesis and vascular endothelial development [36]. It functions as an acyl donor, facilitating LPA acylation, may play a role in MM. LPA is a significant inducer of pro-HB-EGF ectodomain shedding [37], and HB-EGF is also a biomarker for LPA type 1 receptor (LPA1) activation [38]. A recent study demonstrated that MM's BM endothelial cells promote angiogenesis in vivo and in vitro through elevated expression of HB-EGF and EGFR. Inhibition of HB-EGF-EGFR signaling was shown to curtail the angiogenic capabilities of endothelial cells and restrict tumor growth in MM mouse models [39]. Moreover, elevated LPA levels are critical in shielding MM cells from apoptosis induced by PIs in MM patients. LPA's interaction with the LPA2 receptor triggers the downstream MEK1/2-ERK1/2 signaling pathway, enhancing mitochondrial oxidative phosphorylation (OXPHOS), reducing proteasome activity, and improving protein folding in the endoplasmic reticulum, which contributes to MM's resistance to PIs. Utilizing LPA or the LPAR2 receptor as therapeutic targets may enhance the effectiveness of PIs [40]. Whether the interaction between lipid and protein mentioned above promotes the occurrence and progression of MM, further research is needed to clarify the specific mechanism of action.

Anemia is a prevalent symptom in multiple myeloma (MM), affecting approximately 73% of patients at diagnosis [41]. While anemia is often attributed to hematopoietic suppression in MM patients, it can occur even with minimal malignant plasma cell infiltration in the bone marrow. The complex pathophysiological mechanisms underlying anemia in MM remain largely unknown.

In our current study, erythroblasts displayed the highest LMES value and strongly correlated with most of the 50 hallmark pathways, which suggests that abnormal lipid metabolism in erythroid precursors may be a reason for anemia in MM. A recent study published in BLOOD highlighted that PHOSPHO1, an enzyme responsible for the hydrolysis of choline phosphate to choline, is significantly upregulated during the final phase of erythropoiesis in both humans and mice. This upregulation accelerates the breakdown of choline phosphate and its precursor, phosphatidylcholine. The inhibition of phosphatidylcholine and phosphocholine metabolism from PHOSPHO1 knockdown lowers glycine and serine levels, forcing cells to depend on glycolysis for amino acid production. This leads to inadequate ATP production and aberrant lipid accumulation, adversely affecting erythropoiesis during the final differentiation stage [42]. Moreover, ferroptosis plays a crucial role in the physiological processes of embryonic erythropoiesis and erythrocyte senescence [43, 44]. It has been demonstrated that activation of the LPA3 receptor can rescue elastin-induced ferroptosis and erythropoiesis defects by inhibiting lipid oxidation and iron accumulation in erythroleukemia K562 cells [45]. Thus, abnormal lipid metabolism in erythrocytes may impact their maturation and differentiation, offering new insights into the mechanisms underlying MM-associated anemia.

## Conclusions

Consequently, understanding abnormal lipid metabolism-related gene in malignant PCs and the BMM of MM patients is crucial. This knowledge will aid in targeting lipid metabolism reprogramming, restoring immune homeostasis, and improving the prognosis of MM patients.

## Limitations of Study

This study is subject to several limitations. It relies on data extracted from public databases, which allows for the identification of potential correlations between gene expression patterns and MM. However, these correlations are purely indicative. Establishing concrete causative relationships and delineating specific mechanisms of action require comprehensive experimental validation. This underscores the need for further in-depth experimental studies to substantiate the initial findings presented here.

## Data Availability

The datasets of this study mainly come from public databases. http://tisch.comp-genomics.org/home/, GSE161801. https://www.ncbi.nlm.nih.gov/geo/, GSE4204 and GSE136400.

## Abbreviations

ACBD6: Acyl-CoA binding domain containing 6
ASCT: Autologous stem cell transplantation
ATF4: Transcription factor 4
AUC: The area under the curve
BMM: Bone marrow microenvironment
BMAT: Bone marrow adipose tissue
BsAbs: Bispecific antibodies
BTZ: Bortezomib
CAR-T: Chimeric antigen receptor T cell
CIBERSORT: Cell-type identification by estimating relative subsets of RNA transcripts
C-index: Concordance index
DEGs: Differentially expressed genes
ELOVL6: Fatty acid elongase6
Enet: Elastic network
EPIC: Estimating the proportion of immune and cancer cells
FATP1: The fatty acid transporter proteins
FFAs: Free fatty acids
FAO: Fatty acid oxidation
GBM: Gradient boosting machine
GEO: Gene Expression Omnibus database
GO: Gene ontology
GSEA: Gene set enrichment analysis
IPS: Immunophenoscore
KEGG: Kyoto encyclopedia of genes and genomes
LASSO: Least absolute shrinkage and selection operator
LMES: Lipid metabolism-related gene enrichment scores
LMR-DEGs: Differential expression genes related with lipid metabolism
LMRGs: Lipid metabolism-related genes
LMRPgenes: Lipid metabolism-related genes with differential expression associated with MM prognosis
LPA: Lysophosphatidic acid
LPC: Lysophosphatidylcholine
mAbs: Monoclonal antibodies
MCPcounter: Microenvironment cell populations-counter
MHC: Major histocompatibility complex
MM: Multiple myeloma
PCA: Principal components analysis
PCs: Plasma cells
PHD3: Proline hydroxylase-3
PIs: Proteasome inhibitors
plsRcox: Partial least squares regression for cox
ROC: Receiver operating characteristic
RRMM: Relapsed refractory multiple myeloma
RS: Risk scores
RSF: Random forest
SC: Suppressor cell
SREBP1/2: Sterol regulatory element-binding proteins
SuperPC: Supervised principal components
Survival-SVM: Survival support vector machine
TIDE: Tumor immune dysfunction and exclusion
TIMER: Tumor immune estimation resource
UMAP: The unified modular approximation and projection
